# The organizational attributes of HIV care delivery models in Canada: A cross-sectional study

**DOI:** 10.1371/journal.pone.0199395

**Published:** 2018-06-20

**Authors:** Claire E. Kendall, Esther Susanna Shoemaker, Lisa Boucher, Danielle E. Rolfe, Lois Crowe, Marissa Becker, Shabnam Asghari, Sean B. Rourke, Ron Rosenes, Christine Bibeau, Philip Lundrigan, Clare Liddy

**Affiliations:** 1 C.T. Lamont Primary Health Care Research Centre, Bruyère Research Institute, Ottawa, Ontario, Canada; 2 Faculty of Medicine, University of Ottawa, Ottawa, Ontario, Canada; 3 Institute of Clinical and Evaluative Sciences, Toronto, Ontario, Canada; 4 Li Ka Shing Knowledge Institute, St. Michael’s Hospital, Toronto, Ontario; 5 Ottawa Hospital Research Institute, Ottawa, Ontario, Canada; 6 Departments of Medicine, Medical Microbiology and Community Health Sciences, University of Manitoba, Winnipeg, Manitoba, Canada; 7 Department of Family Medicine, Centre for Rural Health Studies, Memorial University of Newfoundland, St. John’s, Newfoundland, Canada; 8 Department of Psychiatry, University of Toronto, Toronto, Ontario, Canada; British Columbia Centre for Excellence in HIV/AIDS, CANADA

## Abstract

HIV treatment in Canada has rapidly progressed with the advent of new drug therapies and approaches to care. With this evolution, there is increasing interest in Canada in understanding the current delivery of HIV care, specifically where care is delivered, how, and by whom, to inform the design of care models required to meet the evolving needs of the population. We conducted a cross-sectional survey of Canadian care settings identified as delivering HIV care between June 2015 and January 2016. Given known potential differences in delivery approaches, we stratified settings as primary care or specialist settings, and described their structure, geographic location, populations served, health human resources, technological resources, and available clinical services. We received responses from 22 of 43 contacted care settings located in seven Canadian provinces (51.2% response rate). The total number of patients and HIV patients served by the participating settings was 38,060 and 17,678, respectively (mean number of HIV patients in primary care settings = 1,005, mean number of HIV patients in specialist care settings = 562). Settings were urban for 20 of the 22 (90.9%) clinics and 14 (63.6%) were entirely HIV focused. Primary care settings were more likely to offer preventative services (e.g., cervical smear, needle exchange, IUD insertion, chronic disease self-management program) than specialist settings. The study illustrates diversity in Canadian HIV care settings. All settings were team based, but primary care settings offered a broader range of preventative services and comprehensive access to mental health services, including addictions and peer support.

## Introduction

With improvements in treatment and acute care, people living with HIV are living longer.[1] Approximately 75,500 Canadians live with HIV [2] and this prevalence is increasing as people being treated live full life spans.[3,4] However, people living with HIV are increasingly experiencing multiple co-morbidities associated with HIV, long-term antiretroviral treatment and aging, such as cardiovascular disease, diabetes, and cancer.[5,6] The impacts of this medical complexity [7] are exacerbated by the negative impact of social determinants of health, the influence of political, social, and economic factors on the health outcomes of individuals.[8] As such, HIV has become a complex chronic condition, requiring a new paradigm of HIV care delivery.

In Canada, HIV clinical care has been managed largely by physicians specialized in HIV or infectious diseases rather than by general primary care providers [9] and most specialist care settings are located in urban areas.[10] Specifically less populated provinces and territories offer HIV services in centralized locations. Previous work has attempted to address findings that HIV specialists have the expertise to treat HIV as a specific condition, but that their care alone might not be sufficient to deliver the expanding comprehensiveness of services required for people aging with HIV.[11–13] Primary care is best equipped to deliver care to people with chronic diseases,[14] but not all primary care providers have the expertise to effectively manage the complexities of HIV treatment.[15–17] In addition, the number of clinicians providing HIV specialist care [18] and enrollment in infectious disease training programs is declining in North America.[19,20] The way HIV care has been organized in Canada may no longer be sustainable, nor meet the needs of the population,[21] with many people with HIV not accessing comprehensive care, despite our universal healthcare system.[22,23]

Given the changing nature and demands on the healthcare system for persons living with HIV, the purpose of this study was to examine the organizational structures and processes of care within the existing HIV focused settings in Canada. By characterizing how many people living with HIV are served, team composition, and extent of resources and services provided in these settings, our results can inform the best practices as care delivery shifts from a predominantly specialist focus towards community based primary care settings.

## Methods

### Design

We conducted a cross-sectional survey between June 2015 and January 2016 to document the organization and composition of HIV clinics and practices in Canada with respect to their geographic locations and settings; the extent of health care and other services offered, and the health human resources and technological resources available. The Ottawa Health Sciences Network Research Ethics Board (protocol #20140649-01H) and the Bruyère Continuing Care Research Ethics Board (protocol #M16-15-011) approved the study.

### Participants/Populations

This study was conducted as part of a large Canadian Institutes of Health Research funded team grant and we used existing relationships of team members in Ontario, Manitoba, and Newfoundland to initiate recruitment. We identified HIV care settings through our clinical networks and by internet searching by province, and used non-probability purposive sampling to identify new potential participating care settings. While we aimed to recruit settings with some established focus on HIV care, we did not restrict our search based on proportion of patients with HIV or sub-population. Additional care settings identified by participants were then contacted via phone and invited to participate.

### Data collection

We developed the Canadian HIV Clinic Survey based on adaptations of validated primary health care surveys; the Patient-Centered Medical Home Assessment (PCMH-A) [24,25], the Canadian Institute for Health Information’s (CIHI) Measuring Organizational Attributes of Primary Health Care Survey,[26] and the Primary Health Care Indicator Framework developed previously by our team.[27] The CIHI survey elements include information on the structural domain of primary care organization, patient populations served, clinic services offered, clinic attributes, technical resources, and location.[28] The Primary Health Care Indicator Framework measures comprehensive community-based primary health care for people with HIV. The survey was sent in an electronic format using FluidSurveys. Participants were instructed at the beginning that submission of the survey indicated consent. Surveys were available in English and French.

### Data analysis

As our intent was to understand comprehensive primary care and specialist care needs of people living with HIV, and given literature demonstrating differences in care delivered by these providers,[29–31] we categorized care settings into two groups: primary care settings, in which a primary care provider (either a family physician and/or a nurse practitioner) is present, and specialist care settings, without an identified primary care provider working on site. We used descriptive statistics to describe the set-up, resources, and clinical services available in HIV care settings.

## Results

**Fig 1:**
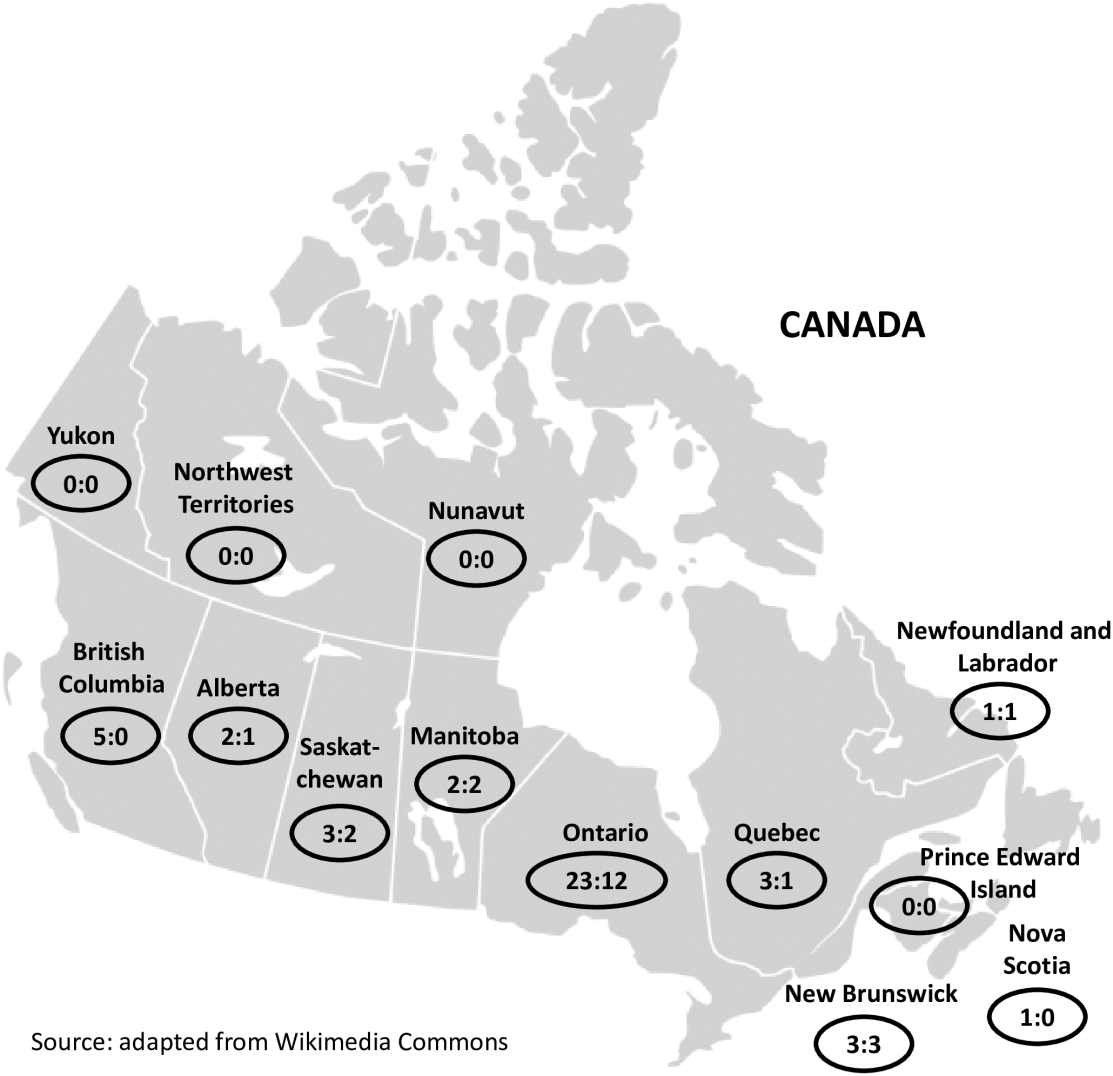
Location and number of invited and participating HIV care settings (# invited: # completed).

Twenty-two of the 43 identified HIV care settings responded (response rate 51%). The majority of the responding settings were located in Ontario (n = 12), with the remaining settings located in New Brunswick (n = 3), Manitoba (n = 2), Saskatchewan (n = 2), Newfoundland (n = 1), Alberta (n = 1), and Quebec (n = 1) (Fig 1). Twenty surveys were completed in English and two in French. Contact was made with clinicians and/or organizations in Prince Edward Island, the Northwest Territories, and Nunavut, but sites within these jurisdictions reported that they did not identify as HIV care settings and that patients from these areas travel to other provinces for HIV care. We were unable to get in touch with a clinician reported to be providing HIV care in Yukon. The participants who completed the survey on behalf of their care settings were in a range of positions, including family or specialist physicians, nurses (registered, practitioners, managers), or clinic leaders (managers, coordinators, directors).

### Populations served

The total number of patients and HIV patients served by the participant HIV care settings was 38,060 and 17,678, respectively (Table 1). Twelve (54.5%) of the 22 clinics were categorized as primary care settings, all of which were located in urban centres. Seven (58.3%) out of 12 primary care settings had only one physical location, two (16.6%) were located in a hospital or university, and three (25%) had multiple locations. Six (50%) primary care settings restricted their services to people living with HIV, one (8.3%) only cared for members of a specific HIV population (i.e. women, men who have sex with men), and one (8.3%) extended their care to family members of people living with HIV. The remaining ten out of 22 (45.5%) participating clinics were defined as specialist care settings, which were predominately located in urban centres (8, 80%) and in a building that is part of a hospital or a university (9, 90%) (Table 2). The majority of specialist care settings only served people living with HIV (8, 80%), while one specialist setting made no restrictions on their patient population (1, 10%) and one restricted services to children under the age of 16 living with HIV (1, 10%) (Table 2).

**Table 1:** Patient population and proportion of patients living with HIV at participating HIV care settings.

	All settings (n = 22)		Primary care setting (n = 12)		Specialist care setting (n = 10)	
	All patients	Patients with HIV	All patients	Patients with HIV	All patients	Patients with HIV
**Total**	38,060	17,678	29,910	12,060	8,150	5,618
**Mean(± sd)**	1,730 (2554)	804 (885)	2,493 (3234)	1,005 (1076)	815 (1209)	562 (619)
**Min**	75	23	160	50	75	23
**Max**	10,000	3,000	10,000	3,000	4,000	1,800

**Table 2:** Patient population, location, payment model, and funding of participating HIV care settings.

	All settings (n = 22) n (%)	Primary care setting (n = 12) n (%)	Specialist care setting (n = 10) n (%)
**Patient Population**			
Any person living with HIV (HIV-specific)	14 (63.6)	6 (50.0)	8 (80.0)
Members of a specific HIV population (i.e. women, men having sex with men)	1 (4.5)	1 (8.3)	0
Infected children < 16 years	1 (4.5)	0	1 (10.0)
Person living with HIV and their family members	1 (4.5)	1 (8.3)	0
Any (general) population	5 (22.7)	4 (33.3)	1 (10.0)
**Rurality**			
Urban	20 (90.9)	11 (91.7)	8 (80.0)
Suburban	2 (9.1)	0	2 (20.0)
Small town/rural	0	0	0
**Location type**			
One physical location	13 (59)	7 (58.3)	6 (60.0)
One physical location linked to affiliated or satellite sites	4 (18)	2 (16.7)	2 (20.0)
Multiple locations, each managed independently	2 (9.1)	2 (16.7)	0
Multiple location with coordinated care and shared administration	3 (13.6)	1 (8.3)	2 (20.0)
**Payment model**			
Fee-for-service	8 (36.4)	4 (33.3)	4 (40.0)
Capitation or roster	1 (4.5)	1 (8.3)	0
Salary	3 (13.6)	2 (16.7)	1 (10.0)
Blended (mix of different models)	8 (36.4)	4 (33.3)	4 (40.0)
Other	1 (4.5)	1 (8.3)	0
Missing	1 (4.5)	-	1 (10.0)
**Funding**			
Targeted program/activity funding or grants	9 (40.9)	7 (58.3)	2 (20.0)
Targeted staffing funding or grants	9 (40.9)	6 (50)	3 (30.0)
Performance-based financial incentives	3 (13.6)	3 (25)	0
Academic research grants	7 (31.8)	4 (33.3)	3 (30.0)
Other	1 (4.5)	1 (8.3)	0
Missing	1 (4.5)	-	1 (10.0)

### Remuneration

An equal number of primary and specialist care settings relied on fee for service physician remuneration (4, 33.3% and 4, 40%) or a blended funding model (4, 33.3% and 4, 40%). All settings reported receiving additional funding through targeted HIV program grants (7, 58.3% primary care versus 2, 20% specialist care), and/or academic research grants (4, 33.3% primary care versus 3. 30% specialist care), among others (Table 2).

### Staffing

**Fig 2:**
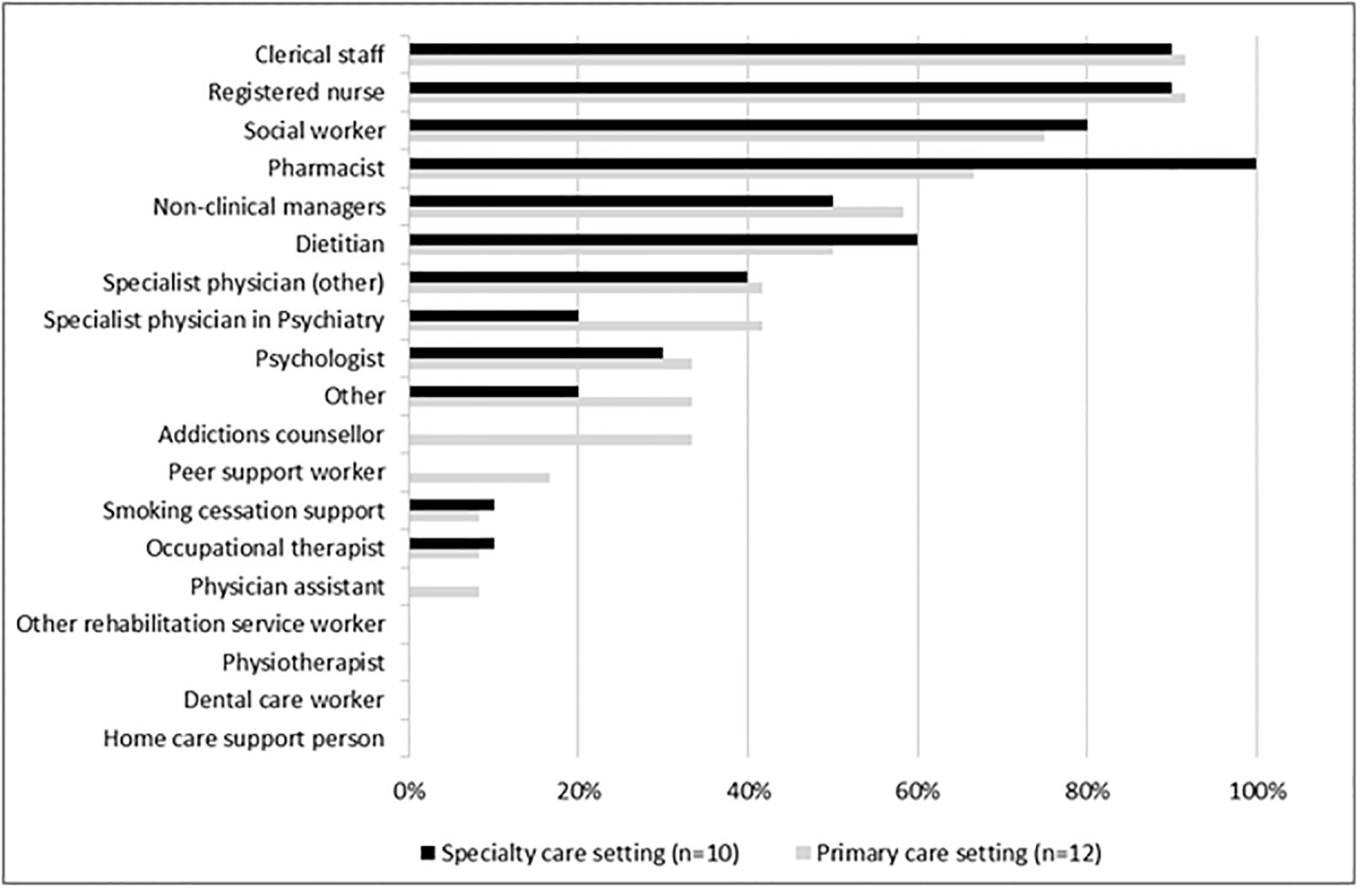
Staffing of primary and specialist care HIV settings. TB = tuberculosis; STI = sexually transmitted diseases; F/U = follow up; IUD = intra uterine device; CDSM = chronic disease self-management; MSK = musculoskeletal.

There was diversity in the availability of allied health practitioners (median Full-Time Equivalent (FTE) per setting 1.25, Interquartile Range (IQR) 1.3). Primary care settings employed more allied health practitioners (median FTE 1.45, IQR 4.1) than specialist settings (median FTE 1.1, IQR 2). A number of settings had staff to provide mental health services, including social workers (17, 77.3%) and psychologists (7, 31.8%), but more primary care settings had psychiatrists (5, 41.7% versus 2, 20%), addictions counsellors (4, 33.3% versus 0), and peer support workers (2, 16.6% versus 0). Dietitians worked at six (50%) primary care settings and six (60%) specialist settings. Rehabilitation services were sparse, with only one (8%) primary care setting and one (10%) specialist setting having an occupational health therapist, and no settings reporting physiotherapy services. All 10 specialist settings had a pharmacist compared to only eight (66.7%) of the primary care settings (Fig 2).

### Health services offered

**Fig 3:**
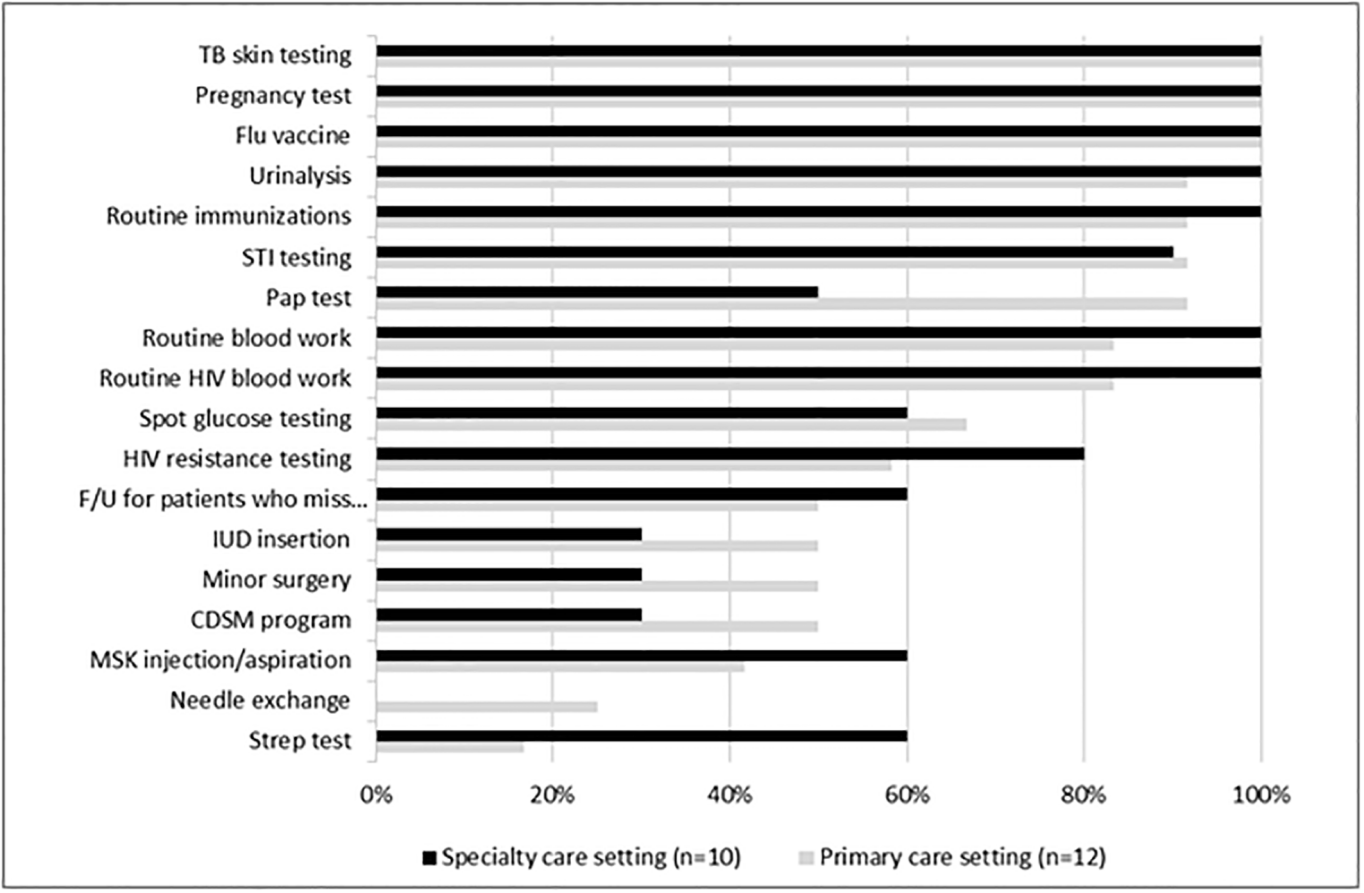
Clinical services available in primary and specialist HIV care settings.

There was considerable variation in the clinical services provided within settings, with primary care settings generally providing a greater range of services. In terms of HIV specific services, most settings offered routine HIV blood work within their facility (10, 83.3% primary care and 10, 100% specialist care) and HIV resistance testing (7, 58.3% primary care and 8, 80% specialist care). The majority of settings offered routine laboratory services, including blood work (10, 83.3% primary care, 10, 100% specialist care), urinalysis (11, 91.7% primary care, 10, 100% specialist care), and all offered pregnancy testing. For preventative health services, the majority of settings provided routine immunizations (11, 91.7% primary care and 10, 100% specialist care) and all offered influenza vaccination. Pap tests were offered in most primary care settings (11, 91.7%) but only in half of the specialist care settings (5, 50%). All settings offered additional public health related services, such as Mantoux skin testing or sexually transmitted infection testing, but only among primary care settings were needle distribution services provided (3, 25%). Extended services for chronic disease self-management programs (6, 50% primary care, 3, 30% specialist care) and procedural services, including minor surgery (6, 50% primary care, 3, 30% specialist care) or intra uterine device insertion (6, 50% primary care, 3, 30% specialist care), were more commonly offered in primary versus specialist settings, although only half of all primary care settings offered such services (Fig 3).

### Information technology resources

All clinics reported being resourced with some form of information technology support and most use computer software to manage appointments (9, 75% primary care, 9, 90% specialist care). Specialist care settings have greater access to on site electronic diagnostic imaging and lab services (10, 100%) than primary care settings (10, 83.3%). Few settings have an electronic system to transmit prescriptions to pharmacies (3, 25% primary care and 1, 10% specialist care). Primary care settings were more likely to report the use of electronic medical records (10, 83.3% primary care versus 5, 50% specialist care), and computerized tools to aid medical decision-making (10, 83.3% primary care versus 3, 30% specialist care). Limited resources are available to support ‘enhanced access’ for patients, such as web-based appointment systems for patients to book appointments (1, 8.3% primary care and 0 specialist care), or an automated electronic option to send appointment reminders to patients (no settings) (Table 3).

**Table 3:** Technological resources available in HIV care settings with and without a primary care provider.

Resource	Primary Care Setting (n = 12) n (%)	Specialist Care Setting (n = 10) n (%)
Information technology support (on site or on call)	12 (100)	10 (100)
Internet access for all staff	11 (92)	10 (100)
Electronic interface to diagnostic imaging & laboratory services	10 (83)	10 (100)
Using EMR	10 (83)	5 (50)
Computerized tools to aid medical decision-making (computerized alerts and recalls, integration of clinical practice guidelines)	10 (83)	3 (30)
Computer software to manage appointments	9 (75)	9 (90)
Unique email addresses for the clinic	5 (42)	3 (30)
An electronic system to transmit prescriptions to pharmacies	3 (25)	1 (10)
A web-based appointment system for patients to book appointments	1 (8)	0
Automated option to send appointment reminders to patients	0	0

## Discussion

We surveyed Canadian HIV care settings providing care to approximately 17,678 people living with HIV, estimated to reflect 23.4% of people living with HIV in Canada.[2] Our study showed that in Canada, HIV care is still very urban centred and that specialist care settings remain very tertiary centre focused. Most settings were team-based, with a wide variety of allied health providers available, such as pharmacists, although primary care settings provided a greater diversity of mental health services including addictions and peer support. Rehabilitation services were notably lacking. While almost all settings had the capacity for HIV specific services, such as CD4 and VL testing, primary care settings additionally offered services related to preventative care, chronic disease self-management programs, and procedural services. Most settings rely on fee for service physician remuneration in addition to HIV/AIDS targeted funding. Finally, all settings had some degree of IT support, although few were using their available technology for full capacity for decision support, patient access, and interfacing with community pharmacies.

Our study builds on the work of others aiming to describe the structures of HIV care setting and how providers work collaboratively to provide care for this complex population.[30] In particular, we were interested in situating our findings within the specialist-primary care dichotomy [9] but we did not find these settings as distinct as we had anticipated. All settings were very team oriented, likely reflecting the needs of the evolving epidemic.[32] Team work has been identified to be important to an HIV care settings’ ability to provide comprehensive services for people living with HIV in both primary and specialist care settings.[33] Settings further performed well on critical HIV care measures, such as the ability to conduct HIV-related blood tests. Pharmacists were present in all specialist settings and extending the reach of pharmacy support can facilitate the management of comorbidities,[34] especially in primary care settings. In addition to offering chronic disease management, primary care settings had a greater range of mental health and addictions services. These services are critical to closing the gaps in mental health care among people living with HIV in Canada,[35] reducing barriers associated with the social determinants of health,[36] and supporting priority populations such as people who use drugs[37] or experience homelessness.[38]

The availability of preventative care services varied in specialist care settings, which speaks to the need to develop and support models of care that bridge HIV specialist with primary care expertise.[39] Effective coordination across primary care and specialist settings is important to improve the comprehensiveness of care for people living with HIV.[33] Such models could include either the expansion of team-based care involving the provision of care by multidisciplinary teams within a single setting, or shared care, such as co-management by multidisciplinary teams across different settings.[30] There is also a potential role for advanced practitioner-based care provided by clinicians who are not physicians, such as nurse practitioners, and who also care primarily for people with HIV.[40]

All of the surveyed settings received additional funding through targeted HIV program grants or research funding. We envision two possible scenarios related to this funding. One is the risk that, as HIV care evolves, the multidisciplinary resourcing of HIV-focused care settings could be scaled back as health systems reorient priorities.[41] Alternatively, the resources within HIV-focused care settings are made available to a broader pool of health care providers serving people living with HIV outside of high volume HIV care settings; thus, extending their reach and optimizing care across the health system.[42,43]

The diverse settings [44] in this study were resourced with information technology but the meaningful uptake of more advanced systems, such as electronic medical records or computerized decision aids, was limited. Harnessing these already available technology systems could contribute to improving care quality, for example, through audit and feedback systems,[45] the facilitation of interprofessional communication,[43] and enhanced patient engagement and access. In addition, robust clinical information systems could contribute to timely measuring and reporting HIV health system performance, in particular related to the HIV care cascade.[27,46]

This study is the first pan Canadian survey of HIV care settings, and is a descriptive study with sampling bias, which therefore does not reflect all types of HIV care across the country. We had an overall response rate to the survey of 51%, were unable to identify HIV specific care settings in the Yukon, Northwest Territories, Nunavut, and Prince Edward Island, and were unable to survey clinics in either British Columbia or Nova Scotia. We were therefore only able to survey clinics in seven provinces, and in some cases only one clinic in a province; however, we do know that in those provinces with smaller populations of people living with HIV, care is very centralized under one HIV program. Twenty of the 22 participating settings were in urban areas, reflecting where most people living with HIV receive care, regardless of whether they live rurally.[2] Some primary care physician groups might have been missed if they do not promote themselves as an HIV care setting. We also know that many people living with HIV in Ontario receive care from physicians who serve a small number of people with HIV and therefore would not be captured through our study. [15–17] The majority of participating settings were from Ontario, which has the highest population and most identified HIV care settings among the provinces.[47] Some clinics in other jurisdictions chose not to participate. In order to provide a more nuanced description of HIV care settings in Canada based on known differences in HIV care delivery, we categorized settings as primary and specialist care. However, while some differences existed in services provided, we found these categories were not as distinct as we had anticipated.

Gaps in current HIV care settings include: broad access to preventative services, comprehensive access to mental health services, access to team based care in non-urban settings, upgrading the use of IT supports to optimize care, and the potential fragility of reliance on targeted HIV programmatic funding. The need for greater involvement and the integration of primary care into the care of people living with HIV is well established,[15–17,41] but HIV shared care remains a fluid concept without a universally accepted definition.[32] Our findings support the need to advance our understanding of how shared care models can ensure comprehensive, timely, and accessible care for people living with HIV.

## Supporting information

S1 Appendix Canadian HIV clinic survey questionnaire. (DOCX)

S1 Dataset Canadian HIV clinic survey data. (XLSX)
