# Supplementary material for: The organizational attributes of HIV care delivery models in Canada: A cross-sectional study
Source: PLoS One. 2018 Jun 20;13(6):e0199395. doi: 10.1371/journal.pone.0199395 (PMC6010295; doi:10.1371/journal.pone.0199395)
Supplement: S1 Appendix — (DOCX) [file pone.0199395.s001.docx]

**Appendix A**

**Canadian HIV Clinic Survey**

Welcome to the Canadian HIV Clinic Survey.

The LHIV Innovation Team thanks you for your interest in this important research.

This survey will take approximately 30-45 minutes to complete. You may complete it in one or more sessions.

This survey is designed to ask questions about how your team works together in caring for persons living with HIV, but also asks for specific human resources data, such as the number of patients in your clinic (HIV and other) and number and type of practitioners.

Please review the Participant Informed Consent Form (on the next page), which provides key information about the study.

The survey consists of two parts:

- **Part 1** is the Patient-Centered Medical Home Assessment (PCMH-A) that has been adapted for the Canadian HIV clinic context.
- **Part 2** has questions about the organization of your clinic. This survey has been adapted in part from the Canadian Institute for Health Information’s’ Measuring Organizational Attributes of Primary Health Care Survey and the Primary Health Care indicator Framework developed by the LHIV Innovation Team (led by, Dr. Sharon Johnston).

Your responses will be automatically saved, and you can return to your survey at any time by clicking on the link in the original email. . When you are satisfied with your responses, click “Submit” to finalize your survey.

Thank you again for your help.

Please contact Lois Crowe at [lcrowe@bruyere.org](mailto:lcrowe@bruyere.org) or toll free at 1-855-561-6891 with any technical issues.

*Note: This survey may not work properly on a cellular phone*.

**PART ONE – Patient-Centered Medical Home Assessment (PCMH-A)-A)**

- **DIRECTIONS** Answer each question from the perspective of one organization (e.g., a practice, clinic, hospital).
- Choose the level that best describes your organization.
- For each question, **click on** the point value that best describes the level of care that currently exists in your organization.
- Each question presents key aspects of patient-centred care. Each aspect is divided into four levels (D to A) showing various stages in development toward a patient-centered medical home.
- The stages are represented by points that range from 1 to 12. The higher point values indicate that the actions described in that box are more fully implemented.

Section 1: PATIENT ROSTERING

| **Components** | **Level D** | **Level C** | **Level B** | **Level A** |
| --- | --- | --- | --- | --- |
| 1. Patients  **Score** | …are not assigned to specific providers.  **1 2 3** | …are assigned to specific providers but patient data are not routinely used by the practice for administrative or other purposes.  **4 5 6** | …are assigned to specific providers and patient data are routinely used by the practice mainly for scheduling purposes.  **7 8 9** | …are assigned to specific providers and patient data are routinely used for scheduling purposes and are continuously monitored to balance supply and demand.  **10 11 12** |
| 2. Registry or patient-level data  **Score** | …are not available to assess or manage care for practice populations.  **1 2 3** | …are available to assess and manage care for practice populations, but only on an ad hoc basis.  **4 5 6** | …are regularly available to assess and manage care for practice populations, but only for a limited number of diseases and risk states.  **7 8 9** | …are regularly available to assess and manage care for practice populations, across a comprehensive set of diseases and risk states.  **10 11 12** |
| 3. Electronic records on individual patients  **Score** | …are not available to practice teams for pre-visit planning or patient outreach.  **1 2 3** | …are available to practice teams but are not routinely used for pre- visit planning or patient outreach.  **4 5 6** | …are available to practice teams and routinely used for pre-visit planning or patient outreach, but only for a limited number of diseases and risk states.  **7 8 9** | …are available to practice teams and routinely used for pre-visit planning and patient outreach, across a comprehensive set of diseases and risk states.  **10 11 12** |
| 4. Reports on care processes or outcomes of care  **Score** | …are not routinely available to providers.  **1 2 3** | …are routinely provided as feedback to providers but not reported externally.  **4 5 6** | …are routinely provided as feedback to providers, and reported externally (e.g. to  patients, other teams or external agencies) but with team identities masked.  **7 8 9** | …are routinely provided as feedback to providers, and transparently reported externally to patients, other teams and external agencies.  **10 11 12** |

Section 2: CONTINUOUS TEAM-BASED HEALING RELATIONSHIPS

| **Components** | **Level D** | **Level C** | **Level B** | **Level A** |
| --- | --- | --- | --- | --- |
| 5. Patients are encouraged to see their own provider and/or practice team  **Score** | …only at the patient’s request.  **1 2 3** | …by the practice administration team, but is not a priority in appointment scheduling.  **4 5 6** | …by the practice administration team and is a priority in appointment scheduling, but patients commonly see other providers because of limited availability or other issues.  **7 8 9** | …by the practice administration team, is a priority in appointment scheduling, and patients usually see their own provider or practice team.  **10 11 12** |
| 6. Non-physician practice team members  **Score** | …play a limited role in providing clinical care.  **1 2 3** | …are primarily tasked with managing patient flow and triage  **4 5 6** | …provide some clinical services such as assessment or self-management support.  **7 8 9** | …perform key clinical service roles that match their abilities and credentials.  **10 11 12** |
| 7. The practice  **Score** | …does not have an organized approach to identify or meet the training needs for providers and other staff.  **1 2 3** | …routinely assesses training needs and assures that staff are appropriately trained for their roles and responsibilities.  **4 5 6** | …routinely assesses training needs, assures that staff are appropriately trained for their roles and responsibilities, and provides some cross training to permit staffing flexibility.  **7 8 9** | …routinely assesses training needs, assures that staff are appropriately trained for their roles and responsibilities, and provides cross training to assure that patient needs  are consistently met.  **10 11 12** |

Section 3: PATIENT-CENTERED INTERACTIONS

| **Components** | **Level D** | | **Level C** | **Level B** | **Level A** |
| --- | --- | --- | --- | --- | --- |
| 8. Assessing patient and family values and preferences  **Score** | …is not done.  **1 2** | **3** | …is done, but not used in planning and organizing care.  **4 5 6** | …is done and providers incorporate it in planning and organizing care on an ad  hoc basis.  **7 8 9** | …is systematically done and incorporated in planning and organizing care.  **10 11 12** |
| 9. Involving patients in decision-making and care  **Score** | …is not a priority.  **1 2** | **3** | …is accomplished by provision of patient education materials or referrals to classes.  **4 5 6** | …is supported and documented by practice teams.  **7 8 9** | …is systematically supported by practice teams trained in decision making techniques.  **10 11 12** |
| 10. Patient comprehension of verbal and written materials  **Score** | …is not assessed.  **1 2** | **3** | …is assessed and accomplished by assuring that materials are at a level and language that patients understand.  **4 5 6** | …is assessed and accomplished by hiring multi-lingual staff, and assuring that both materials and communications are at a level and language that patients understand.  **7 8 9** | …is supported at an organizational level by translation services, hiring multi-lingual staff, and training staff in health literacy and communication techniques (such as closing the loop) assuring that patients know what to do to manage conditions at home.  **10 11 12** |

PART 3: PATIENT-CENTERED INTERACTIONS CONTINUED ON NEXT PAGE

Section 3: PATIENT-CENTERED INTERACTIONS CONTINUED

| **Components** | **Level D** | **Level C** | **Level B** | **Level A** |
| --- | --- | --- | --- | --- |
| 11. Self-management support  **Score** | …is limited to the distribution of information (pamphlets, booklets).  **1 2 3** | …is accomplished by referral to self-management classes or educators.  **4 5 6** | …is provided by goal setting and action planning with members of the practice team.  **7 8 9** | …is provided by members of the practice team trained in patient empowerment and problem-solving methodologies.  **10 11 12** |
| 12. The principles of patient-centered care  **Score** | …are included in the organization’s vision and mission statement.  **1 2 3** | …are a key organizational priority and included in training and orientation.  **4 5 6** | …are explicit in job descriptions and performance metrics for  all staff.  **7 8 9** | …are consistently used to guide organizational changes and measure system performance as well as care interactions at the practice level.  **10 11 12** |
| 13. Measurement of Patient Centered Interactions  **Score** | …is not done or is accomplished using a survey administered sporadically at the organization level.  **1 2 3** | … is accomplished through patient representation on boards and regularly soliciting patient input through surveys.  **4 5 6** | … is accomplished by getting frequent input from patients and families using a variety of methods such as point of care surveys, focus groups and  ongoing patient advisory groups.  **7 8 9** | …is accomplished by getting frequent and actionable input from patients and families on all care delivery issues, and incorporating their feedback in quality improvement activities.  **10 11 12** |

Section 4: ENGAGED LEADERSHIP

| **Components** | **Level D** | **Level C** | **Level B** | **Level A** |
| --- | --- | --- | --- | --- |
| 14. Executive leaders  **Score** | …are focused on short-term business priorities.  **1 2 3** | …visibly support and create an infrastructure for quality  improvement, but do not commit resources.  **4 5 6** | …allocate resources and actively reward quality improvement initiatives.  **7 8 9** | …support continuous learning throughout the organization, review and act upon quality data, and have a long-term strategy and funding commitment to explore, implement and spread quality improvement initiatives.  **10 11 12** |
| 15. Clinical leaders  **Score** | …intermittently focus on improving quality.  **1 2 3** | …have developed a vision for quality improvement, but no consistent process for  getting there.  **4 5 6** | …are committed to a quality improvement process, and sometimes engage teams  in implementation and problem solving.  **7 8 9** | … consistently champion and engage clinical teams in improving patient experience of care and clinical outcomes.  **10 11 12** |
| 16. The organization’s hiring and training processes  **Score** | …focus only on the narrowly defined functions and requirements of each position.  **1 2 3** | …reflect how potential hires will affect the culture and participate in quality improvement activities.  **4 5 6** | …place a priority on the ability of new and existing staff to improve care and create a patient-centered culture.  **7 8 9** | …support and sustain improvements in care through training and incentives focused on rewarding patient-centered care.  **10 11 12** |
| 17. The responsibility for conducting quality improvement activities  **Score** | …is not assigned by leadership to any specific group.  **1 2 3** | …is assigned to a group without committed resources.  **4 5 6** | …is assigned to an organized quality improvement group who receive dedicated resources.  **7 8 9** | …is shared by all staff, from leadership to team members, and is made explicit through protected time to meet and specific resources to engage in QI.  **10 11 12** |

Section 5: QUALITY IMPROVEMENT (QI) STRATEGY

| **Components** | **Level D** | **Level C** | **Level B** | **Level A** |
| --- | --- | --- | --- | --- |
| 18. Quality improvement activities  **Score** | …are not organized or supported consistently.  **1 2 3** | …are conducted on an ad hoc basis in reaction to specific problems.  **4 5 6** | …are based on a proven improvement strategy in reaction to specific problems.  **7 8 9** | …are based on a proven improvement strategy and used continuously in meeting organizational goals.  **10 11 12** |
| 19. Performance measures  **Score** | …are not available for the clinical site.  **1 2 3** | …are available for the clinical site, but are limited in scope.  **4 5 6** | …are comprehensive – including clinical, operational, and patient experience measures – and available for the practice, but not for individual providers.  **7 8 9** | …are comprehensive – including clinical, operational, and patient experience measures – and fed back to individual providers.  **10 11 12** |
| 20. Quality improvement activities are conducted by  **Score** | …a centralized committee or department.  **1 2 3** | …topic specific QI committees.  **4 5 6** | …all practice teams supported by a QI infrastructure.  **7 8 9** | …practice teams supported by a QI infrastructure with meaningful involvement of patients and families.  **10 11 12** |
| 21. An Electronic Health Record that is Meaningful-Use certified  **Score** | …is not present or is being implemented.  **1 2 3** | … is in place and is being used to capture clinical data.  **4 5 6** | …is used routinely during patient encounters to provide clinical decision support and to share data with patients.  **7 8 9** | … is also used routinely to support population management and quality improvement efforts.  **10 11 12** |

Section 6: ENHANCED ACCESS

| **Components** | **Level D** | **Level C** | **Level B** | **Level A** |
| --- | --- | --- | --- | --- |
| 22. Appointment systems  **Score** | …are limited to a single office visit type.  **1 2 3** | …provide some flexibility in scheduling different visit lengths.  **4 5 6** | … provide flexibility and include capacity for same day visits.  **7 8 9** | …are flexible and can accommodate customized visit lengths, same day visits, scheduled follow-up and multiple provider visits.  **10 11 12** |
| 23. Contacting the practice team during regular business hours  **Score** | …is difficult.  **1 2 3** | …relies on the practice’s ability to respond to telephone messages.  **4 5 6** | …is accomplished by staff responding by telephone within the same day.  **7 8 9** | …is accomplished by providing a patient a choice between email and phone interaction, utilizing systems which are monitored for timeliness.  **10 11 12** |
| 24. After-hours access  **Score** | ...is not available or limited to an answering machine.  **1 2 3** | …is available from a coverage arrangement without a standardized communication protocol back to the practice for urgent problems.  **4 5 6** | …is provided by coverage arrangement that shares necessary patient data and provides a summary to the practice.  **7 8 9** | …is available via the patient’s choice of email, phone or  in-person directly from the practice team or a provider closely in contact with the team and patient information.  **10 11 12** |

Section 7: CARE COORDINATION

| **Components** | **Level D** | **Level C** | **Level B** | **Level A** |
| --- | --- | --- | --- | --- |
| 25. Medical and surgical specialty services  **Score** | …are difficult to obtain reliably.  **1 2 3** | …are available from community specialists but are neither timely nor convenient.  **4 5 6** | …. are available from community specialists and are generally timely and convenient.  **7 8 9** | …are readily available from specialists who are members of the care team or who work in an organization with which the  practice has a referral protocol or agreement.  **10 11 12** |
| 26. Behavioral health services  **Score** | …are difficult to obtain reliably.  **1 2 3** | …are available from mental health specialists but are neither timely nor convenient.  **4 5 6** | …are available from community specialists and are generally timely and convenient.  **7 8 9** | …are readily available from behavior health specialists who are onsite members of the care team or who work in a community organization with  which the practice has a referral protocol or agreement.  **10 11 12** |
| 27. Patients in need of specialty care, hospital care, or supportive community-based resources  **Score** | …cannot reliably obtain needed referrals to providers with whom the practice has a relationship.  **1 2 3** | …obtain needed referrals to partners with whom the practice has a relationship.  **4 5 6** | …obtain needed referrals to providers with whom the practice has a relationship and relevant information is communicated  in advance.  **7 8 9** | …obtain needed referrals to providers with whom the practice has a relationship, relevant information is communicated in advance, and timely follow-up after the visit occurs.  **10 11 12** |

PART 7: CARE COORDINATION CONTINUED ON NEXT PAGE

Section 7: CARE COORDINATION CONTINUED

| **Components** | **Level D** | **Level C** | **Level B** | **Level A** |
| --- | --- | --- | --- | --- |
| 28. Follow-up by the primary care practice with patients seen in the Emergency Room or hospital  **Score** | ...generally does not occur because the information is not available to the primary care team.  **1 2 3** | …occurs only if the ER or hospital alerts the primary care practice.  **4 5 6** | …occurs because the primary care practice makes proactive efforts to identify patients.  **7 8 9** | …is done routinely because the primary care practice has arrangements in place with the ER and hospital to both track these patients and ensure that follow-up is completed within a few days.  **10 11 12** |
| 29. Linking patients to supportive community-based resources  **Score** | …is not done systematically.  **1 2 3** | …is limited to providing patients a list of identified community resources in an accessible format.  **4 5 6** | …is accomplished through a designated staff person or resource responsible for connecting patients with community resources.  **7 8 9** | …is accomplished through active coordination between the health system, community service agencies and patients and accomplished by a designated staff person.  **10 11 12** |
| 30. Test result and care plans  **Score** | …are not communicated to patients.  **1 2 3** | …are communicated to patients based on an ad hoc approach.  **4 5 6** | …are systematically communicated to patients in a way that is convenient to the practice.  **7 8 9** | …are systematically communicated to patients in a variety ways that are convenient to patients.  **10 11 12** |

Section 8: ORGANIZED, EVIDENCE-BASED CARE

| **Components** | **Level D** | **Level C** | **Level B** | **Level A** |
| --- | --- | --- | --- | --- |
| 31. Comprehensive, guideline-based information on prevention or chronic illness treatment  **Score** | …is not readily available in practice.  **1 2 3** | …is available but does not influence care.  **4 5 6** | …is available to the team and is integrated into care protocols and/or reminders.  **7 8 9** | …guides the creation of tailored, individual-level data that is available at the time of the visit.  **10 11 12** |
| 32. Visits  **Score** | …largely focus on acute problems of patient.  **1 2 3** | …are organized around acute problems but with attention to ongoing illness and prevention needs if time permits.  **4 5 6** | …are organized around acute problems but with attention to ongoing illness and prevention needs if time permits. The practice also uses subpopulation reports to proactively call groups of  patients in for planned care visits.  **7 8 9** | …are organized to address both acute and planned care needs. Tailored guideline-based information is used in team huddles to ensure all outstanding patient needs are met at each encounter.  **10 11 12** |
| 33. Care plans  **Score** | …are not routinely developed or recorded.  **1 2 3** | …are developed and recorded but reflect providers’ priorities only.  **4 5 6** | …are developed collaboratively with patients and families and include self-management and clinical goals, but they are not routinely recorded or used to guide subsequent care.  **7 8 9** | …are developed collaboratively, include self-management and clinical management goals, routinely recorded and guide care at every subsequent point  of service.  **10 11 12** |
| 34. Clinical care management services for high risk patients  **Score** | …are not available.  **1 2 3** | …are provided by external care managers with limited connection to practice.  **4 5 6** | …are provided by external care managers who regularly communicate with the  care team.  **7 8 9** | …are systematically provided by the care manager functioning as a member of the practice team, regardless of location.  **10 11 12** |

**Canadian HIV Clinic Survey Part Two**

In this part of the survey, we are asking questions to understand specific ways that your clinic is organized. Please provide as much information as you can.

Choose one answer per question, unless otherwise indicated.

**Section One: Population Served**

1. Which statement best represents the population that your clinic serves? Please choose one answer.

- Any person living with HIV
- Members of a specific HIV population (i.e. women, MSM)
- Infected children under the age of 16
- Service includes family members of persons living with HIV
- No restriction based on population

1. How many patients are registered at your clinic? Please provide your best estimate. _______
2. Of all the patients you serve, how many have HIV? Please provide your best estimate. _______
3. Are there any comments you would like to make about the population your clinic serves?

_______________________________________________________________________

**Section Two: Clinic Attributes**

1. Please identify the type of practitioners working at your clinic. Please estimate the amount of time in Full-Time Equivalent (FTE) hours each group is working each week.

Note: For each practioner type, the total FTE should not be higher than the total number of people who hold that position.

If you do not have a certain type of practioner working in your clinic please select “Not Applicable”.

| **Type of Practitioner** | **Not Applicable** | **How many of each practitioner type work in your clinic?** | **What is the total FTE in your clinic for each practitioner type?**  (Note: 1.0 FTE = 35 hours per week or more) |
| --- | --- | --- | --- |
| Family physician |  |  |  |
| Nurse practitioner |  |  |  |
| Registered nurse |  |  |  |
| Physician assistant |  |  |  |
| Specialist physician (infectious disease) |  |  |  |
| Specialist physician (other) |  |  |  |
| Psychiatrist |  |  |  |
| Psychologist |  |  |  |
| Pharmacist |  |  |  |
| Addictions counsellor |  |  |  |
| Social Worker |  |  |  |
| Peer support worker |  |  |  |
| Dietitian |  |  |  |
| Home care support person |  |  |  |
| Dental care worker |  |  |  |
| Occupational or Physiotherapist |  |  |  |
| Rehabilitation service worker |  |  |  |
| Smoking cessation support worker |  |  |  |
| Clerical staff |  |  |  |
| Non-clinical managers |  |  |  |
| Other (please specify) |  |  |  |

Are there any comments you would like to make about the types of practioners at your clinic?

**Section Three: Clinic Services Offered**

1. For persons living with HIV that you serve, which of the following clinical tests and services are available on site?

| **Tests/Immunizations** | **Yes** | **No** |
| --- | --- | --- |
| Routine blood work |  |  |
| Routine HIV blood work (CD4, VL) |  |  |
| HIV resistance testing |  |  |
| Rapid streptococcal test (strep test) |  |  |
| Cervical smear (pap test) |  |  |
| Routine immunizations |  |  |
| Influenza (seasonal flu) vaccination |  |  |
| Pregnancy test |  |  |
| Urinalysis |  |  |
| Mantoux (TB) skin testing |  |  |
| Sexually transmitted infection testing |  |  |
| Spot glucose testing (glucoscan) |  |  |
| **Services/Procedures** |  |  |
| Needle exchange |  |  |
| Suture/minor surgery |  |  |
| Musculoskeletal injection/aspiration |  |  |
| IUD insertion |  |  |
| Chronic disease self-management program |  |  |
| Established process and resources to follow up on patients who miss appointments |  |  |

Are there any comments you would like to make about the clinic tests and services available at your clinic?

**Section Four: Technical Resources**

1. In your clinic, do you have an electronic medical record system?

- Yes ❑ Planned, but not yet implemented ❑ Not Sure ❑ No

If yes or planned:

Which EMR system do you use?

_________________________________________________________

Are there any comments you would like to make about using an EMR system?

_________________________________________________________

1. In your clinic, do you use?

|  | **Yes** | **No** | **Planned** | **Not sure** |
| --- | --- | --- | --- | --- |
| Internet access for all staff |  |  |  |  |
| Computerized tools to aid medical decision-making (computerized alerts and recalls, integration of clinical practice guidelines) |  |  |  |  |
| Electronic interface to diagnostic imaging & laboratory services |  |  |  |  |
| An electronic system to transmit prescriptions to pharmacies |  |  |  |  |
| A web-based appointment system for patients to book appointments |  |  |  |  |
| Information technology support (on site or on call) |  |  |  |  |
| Computer software to manage appointments |  |  |  |  |
| Unique email addresses for the clinic (i.e. name@myclinic.ca) |  |  |  |  |
| Automated option to send appointment reminders to patients |  |  |  |  |

**No**

Are there any comments you would like to make about the technical resources in your clinic?

____________________________________________________________________________

**Section Five: About you and your Clinic**

1. How would you characterize the locale for your clinic?

❑ City ❑ Suburb ❑ Small town ❑ Rural

1. Where is your clinic located?

- In a building owned by the physicians or of which they are shareholders
- In rented offices in a commercial building for health professionals
- In rented offices in a commercial building for any type of business
- In an establishment that is part of the publicly funded health network (hospital, etc.) or university
- Other *(please specify)*: __________________________________________________

1. What type of physical locations are considered as part of your clinic?

- One physical location
- One physical location but linked to affiliated or satellite sites
- More than one location, but each location is managed independently
- More than one location with coordination of care and administrative activities between sites

1. What funding arrangement best describes the payment model for physicians in your clinic?

- Fee-for-service
- Capitation or roster
- Salary (hourly rate, sessional payment, contract)
- Blended model (mix of different payment models)
- Other *(please specify)*: __________________________________________________

1. Does your clinic receive other types of funding from:

|  | **Yes** | **No** |
| --- | --- | --- |
| Targeted program/activity funding/grants |  |  |
| Targeted staffing funding/grants |  |  |
| Performance-based financial incentives |  |  |
| Academic research grants |  |  |
| Other *(please specify)*: |  |  |

Are there any comments you would like to make about your clinic location or funding?

___________________________________________________________________________

**Briefly describe the process you used for parts 1 and 2 to fill out the survey** (e.g., each team member filled out a separate form and reached consensus in a face-to-face meeting; filled out by the team leader in consultation with other team members as needed).

On behalf of the LHIV Innovation Team, we want to thank you for taking the time to complete this important survey.

With your help and insights, we hope to make important recommendations to improve care for people living with HIV.

**Additional Request**

We may wish to conduct confidential interviews with you or members of your team after the survey analysis is complete. The purpose of these interviews would be to better understand approaches to the changes needed in caring for persons living with HIV.

Online Instructions

If you choose to provide your contact information, click on the link that appears. If you select **Yes, you will be directed to a new page**. By clicking on the link you will not lose any of the information that you have already entered into the survey. Once you have completed the contact form, please make sure you return to submit the survey.

The information you provide on this contact form and during any follow-up interviews will not be linked to the survey responses already provided.

If you do not wish to provide your contact information, simply select **No** below.

Do you give your permission for us to contact you for separate, confidential interviews if needed?

❑ Yes ❑ No

If they choose yes, there will be a link here with a pop up or automatic email (depending on the survey platform’s capacity) that will direct them to answer these questions in a separate document or an automatic email will be sent asking them to complete a form with this information to be returned to the research team.

Please contact Lois Crowe at [lcrowe@bruyere.org](mailto:lcrowe@bruyere.org) or toll free at 1-855-561-6891 if you have any questions about the survey administration or technical issues.

**Thank you!**

Please press SUBMIT when you are satisfied with your answers.

Paper Instructions

If you choose to provide your contact information, please complete the separate Permission to Contact Form. If you choose not to provide your information, you do not need to complete the form.

The Permission to Contact Form is a separate page that will not be kept with your survey results, so the information you provide on this contact form and during any follow-up interviews will not be linked to the survey responses.

Alternatively, please first copy and paste the following table into an email, then fill in your contact information and send it to [lcrowe@bruyere.org](mailto:lcrowe@bruyere.org)**.**

**Permission to Contact Form**

**STUDY TITLE**

A mixed methods study to characterize the organizational attributes and patient-centred medical home features of HIV care settings in Canada. (OHSN #20140649-01H, Bruyère #M16-15-011)

| Thank you for agreeing to be contacted for separate and confidential interviews.  Please tell us the best way to contact you. Please note that the information you provide will be kept confidential and that your name or the clinic name will never be released in any report or publication. Any contact information you provide will not be linked to your survey responses.  *Indicates a Required Field  Clinic Name: ______________________________________________________  Clinic Address: ____________________________________________________  Main Clinic Location (street address, city, province, postal code): ____________  ________________________________________________________________  *Your Name (the name of the person completing the survey): _______________  Your position in the clinic:_________________________  Your phone number: ____________________________  Your alternate phone number: _____________________  Your fax number: _______________________________  *Your email address:_____________________________  *I prefer to be contacted by ❑ phone ❑ email ❑ fax |
| --- |
